# Why do mothers encourage their children to control their weight? A cross-sectional study of possible contributing factors

**DOI:** 10.1186/1471-2458-14-450

**Published:** 2014-05-13

**Authors:** Anja C Schreiber, Dorothea Kesztyüs, Tamara Wirt, Nanette Erkelenz, Susanne Kobel, Jürgen M Steinacker

**Affiliations:** 1 Division of Sports and Rehabilitation Medicine, Department of Internal Medicine II, Ulm University Medical Centre, Frauensteige 6, 89075 Ulm, Germany

**Keywords:** Parental behaviour, Mothers, Children, Overweight

## Abstract

**Background:**

Mothers encouraging their children to control their weight is problematic as it is associated with children’s body dissatisfaction and weight concerns as well as further weight gain. The aim of this study was to identify factors in children and mothers associated with mothers encouraging their children to control their weight and possible gender differences therein.

**Methods:**

Cross-sectional questionnaire data was available from 1658 mothers of primary school children (mean age 7.1 ±0.6 years, 50.4% boys) participating in the Baden-Württemberg Study. Children’s body weight and height were measured in a standardised manner. Logistic regressions were computed separately for boys and girls, adjusted odds ratios (OR) and 95% confidence intervals (CI) from the final model are reported.

**Results:**

29% of children were encouraged by their mothers, girls (32.4%) significantly more often than boys (25.6%). Child BMI (girls OR 1.77, CI 1.57 to 1.99; boys OR 1.88, CI 1.66 to 2.13), and child migration background (girls OR 2.14, CI 1.45 to 3.16; boys OR 1.60, CI 1.07 to 2.37) were significantly associated with encouragement by mothers. For girls, maternal body dissatisfaction (OR 1.59, CI 1.10 to 2.30) and maternal perception of a low influence on health (OR 0.51, CI 0.29 to 0.89) were also significantly associated with maternal encouragement. For boys, this was true of mothers self-efficacy to influence their children’s physical activity (OR 0.58, CI 0.40 to 0.85).

**Conclusion:**

Different factors are associated with mothers encouraging boys and girls to control their weight. Identifying correlates and underlying processes of maternal encouragement can inform preventive measures targeting weight and eating related problems in children.

## Background

The high prevalence of childhood obesity has led to frequent discussions of healthy eating and weight in children as well as their conditions and consequences. Parents are considered to play an important role in shaping their children’s weight related behaviours and attitudes [1]. This is especially true for mothers who still carry most of the burden of child care. Thus, parents are often targeted in childhood obesity prevention efforts and related public health campaigns. But as O’Dea points out there is always the possibility of "perfectly reasonable and well-intentioned health messages being partially misconstrued or misunderstood" [2], p. 259. Health education messages regarding overweight focusing on weight control, for example, may unintentionally lead to parental behaviour that promotes body image or weight concerns, and even further weight gain in children.

There is evidence that mothers’ restrictive feeding practices like limiting access to certain food may lead to weight gain and the development of problematic eating behaviours [3-5]. Research on body image and weight concerns in children and adolescents has shown, that parental encouragement to control weight and even just weight related comments are associated with weight concerns and disordered eating behaviour in children [6-8].

In the light of negative side effects of such strategies an insight into possibly associated factors may be important for the development of preventive measures targeting weight and eating related problems in children.

Presuming that parents are more likely to engage in areas of life of concern to themselves, as has been previously proposed [9], it is reasonable to assume that mothers are more likely to encourage their children to control their weight if their child’s weight or their own weight are of concern to them. Indeed, Francis & Birch found that encouragement to lose weight was related to mothers’ own preoccupation with weight and eating [7]. Other studies found similar associations with parental body dissatisfaction and weight concerns [10,11]. In accordance with the above presumption studies have also shown that maternal encouragement to control weight is related to child’s body mass and mothers’ concern about or recognition of their child’s overweight [12,13].

Given that there are gender differences in regards to societal body ideals, e.g. the higher pressure to be thin for females, it seems reasonable to assume that girls are more often encouraged to control their weight than boys and that there are also different underlying motivations for mothers to influence weight-related behaviours of their sons and daughters, respectively. While some studies did not find gender differences in the frequency of maternal encouragement to control weight, others report more parental messages regarding weight to daughters than to sons [6,11,14,15]. Additionally, data from a study of mothers of 5-8 year old children support the assumption of different determinants of maternal control over children’s eating with mothers restraint playing a role in monitoring girls but not boys [16]. Nonetheless, there is still a paucity of research into possible gender differences since most studies only study girls or don’t look at boys and girls separately.

Therefore, the current study aims to gain more insight into factors related to maternal encouragement to control weight in a large non-clinical sample from socio-economically diverse backgrounds. Moreover, the study means to shed light on possible gender differences between processes underlying that maternal behaviour by looking at possibly related variables for boys and girls separately. We expected heavier children as well as girls to be encouraged more often to control their weight. Additionally, we presumed a higher risk of encouragement to control their weight in children with mothers who are dissatisfied with their own body. Assuming its potential use as a maternal weight management strategy encouragement to control weight may also be associated with other attitudes and behaviours related to weight and health. Thus, we explored whether maternal encouragement to control weight was also associated with mothers health behaviours, their health consciousness as well as their confidence to be able to influence health in general and weight related behaviours of their children in particular.

## Methods

### Design and participants

This work presents cross-sectional results from baseline measurements of the Baden-Württemberg Study in south-west Germany. The main goal of the Baden-Württemberg Study was to evaluate a school-based health promotion programme with a focus on overweight prevention for primary school children. Details about the programme and the evaluation study have already been published elsewhere [17,18]. The study was approved by the ethics committee of Ulm University (Application No. 126/10) and is registered at the German Clinical Trials Register, Freiburg University, Germany (DRKS-ID DRKS00000494).

**Figure 1:**
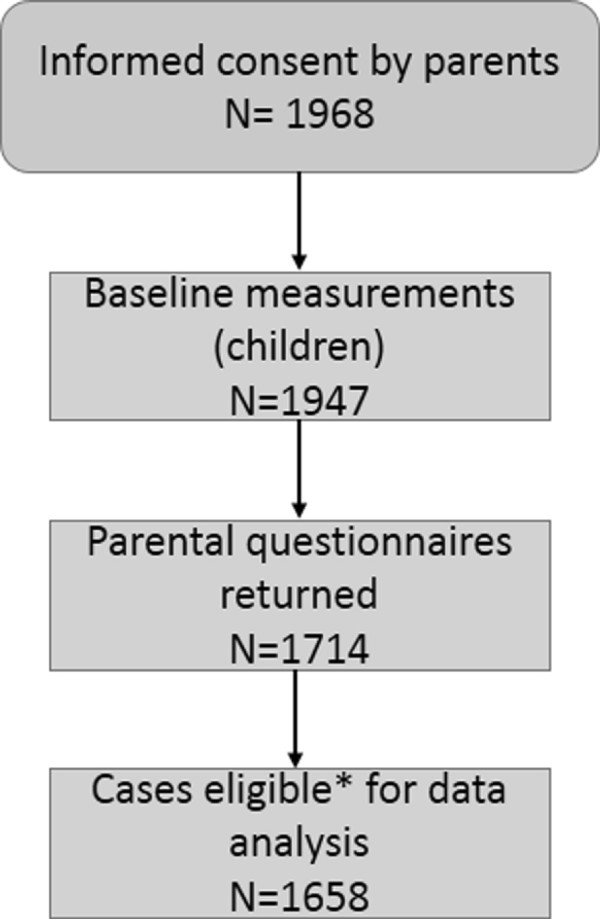
**Overview of sample size.** *Cases with complete data on encouragement by mothers, child gender and child age were considered eligible for analysis.

A total of 1968 parents gave their written informed consent, representing 62.3% of the 157 classes participating in the study at baseline. In autumn 2010, 1947 children in first and second grade were examined on site and parents received a questionnaire. Questionnaire data were available of 1714 participants (see Figure 1).

### Measures

All measures presented in this article with the exception of children’s weight and height were assessed via parental questionnaire.

#### Demographics

Level of education of mothers and fathers was assessed and categorised according to the CASMIN classification [19]. Maternal level of education was then dichotomized into tertiary level education (= college or university degree) and elementary/intermediate level education. Migration background of the child was defined as either at least one parent born abroad or at least one parent having spoken in a language other than German with the child in its first years of life.

#### Body mass index

Children’s weight and height were measured by trained staff with calibrated flat scales and stadiometers (Seca, Germany). Mothers provided their weight and height using the questionnaire. Body-mass-index (BMI) was calculated as weight divided by height squared (kg/*m*^2^).

#### Maternal encouragement and maternal self-efficacy to influence their child’s health behaviours

The frequency of mothers encouraging their children to control their weight (maternal encouragement) was assessed on a 5-point rating scale (*How often do you encourage your child to control its weight?*). Further, maternal self-efficacy to influence their children’s health behaviour in three different domains was assessed on 4-point rating scales. Self-efficacy refers to a person’s belief about their capability to successfully perform a particular behaviour or in a particular situation. The assessed domains of maternal self-efficacy to influence their children’s health behaviour were TV consumption (*I manage to ensure my child does not watch too much TV.*), soft drink consumption (*I manage to ensure my child does not drink too much sugary beverages.*), and physical activity (*I manage to ensure my child is sufficiently physical active.*).

#### Maternal health-related behaviours and cognitions

Mothers reported whether they were currently smoking and whether they were actively engaging in sports. Additionally, they were asked to rate their health consciousness (*How much do you take care of your health in general?*) and their perceived degree of influence on health (*How much can one do to maintain or improve one’s health?*) on 4-point rating scales. Health consciousness refers to the amount of concern an individual shows for the healthiness of their lifestyle. The perceived degree of influence on health refers to the respondent’s belief about to which degree an individual’s behaviour can have a positive impact on their health status. Mothers’ dissatisfaction with their bodies (maternal body dissatisfaction) was also assessed (*Do you think you are much too thin, too thin, alright, too fat, much too fat?*).

### Data analyses

Inclusion criteria for the analysis were complete data on encouragement by mothers, child gender and child age. This resulted in an overall sample of n=1658 (see Figure 1 for an overview of the study sample size). Due to skewed and not classifiable distribution several variables were recoded for the analyses. The outcome variable maternal encouragement to control weight was dichotomised into "never encourages child" and "encourages child". Similarly, maternal health consciousness, perception of degree of influence on health and self-efficacy to influence child’s health behaviour (all three domains) were dichotomised at the median. This resulted in variables differentiating between a high degree and a low degree of health consciousness, perceived degree of influence on health and self-efficacy, respectively. For the logistic regression analyses a low degree in those variables was chosen as reference category. Maternal body dissatisfaction was dichotomised into "is dissatisfied" versus "is not dissatisfied". Since the focus of this study lays upon concerns regarding overweight the 53 mothers who perceived themselves as too thin or much too thin were excluded from the analysis.

Gender differences were tested using student’s t-test for continuous variables (BMI and age) and *χ*^2^ test for categorical data. Univariate logistic regressions were performed to identify factors associated with maternal encouragement. Variables with p-values below.10 were then included in multivariate regression analyses using stepwise backward elimination to verify associated factors. Finally, to compute odds ratios and 95% confidence intervals adjusted for child’s age and maternal education level multivariate regressions with enter method analysis were performed. All regression analyses were computed for boys and girls separately. Due to missing values sample size may vary for different analyses. All data analyses were carried out with IBM SPSS 19.00.02.

## Results

### Sample description

The baseline characteristics of the sample (835 boys and 823 girls) and the studied variables are shown in Table 1. Boys and girls differ significantly only in the number of mothers with a high self-efficacy to increase their child’s physical activity (*χ*^2^(1)=5.90, p=.015). Children’s weight status was computed using German reference percentiles [20], accordingly the prevalence in this sample was 7.4% for underweight, 84.1% for normal weight, and 8.5% for overweight. Mothers’ mean age at the time of data collection was 37.4 years (SD=5.1, range: 24-52 years) and the majority (65.6%) reported an annual household income between 21,000 and 48,000 Euro.

**Table 1:** Sample characteristics

	**Missing values**	**Boys**	**Girls**	**Total n=1658**
**Child characteristics**				
Age, *years* [mean (sd)]	0	7.1 (0.6)	7.0 (0.6)	7.1 (0.6)
Migration background, *yes* (%)	54	30.1	31.9	31.0
Child’s BMI, kg/ *m*^2^ [mean (sd)]	48	16.0 (2.0)	15.9 (2.1)	16.0 (2.1)
**Maternal characteristics**				
Maternal BMI, kg/ *m*^2^ [mean (sd)]	109	24.1 (4.5)	24.2 (4.5)	24.1 (4.5)
Tertiary education level (%)	17	19.6	18.8	19.2
Currently smoking, *yes* (%)	38	20.9	21.1	21.0
Active in sports, *yes* (%)	103	57.1	59.7	58.7
Body dissatisfaction, *yes* (%)	84	49.6	49.9	49.7
Health consciousness, *high* (%)	11	58.5	58.8	58.7
Perceived degree of influence				
on health, *high* (%)	29	88.6	88.8	88.7
Self-efficacy to influence child’s				
- TV consumption, *high* (%)	19	58.5	58.5	58.5
- soft drink consumption, *high* (%)	18	60.6	61.8	61.2
- physical activity, *high* (%)	21	57.0*	51.0	54.0

*Note.* sd = standard deviation; * p<.05.

### Maternal encouragement

**Figure 2:**
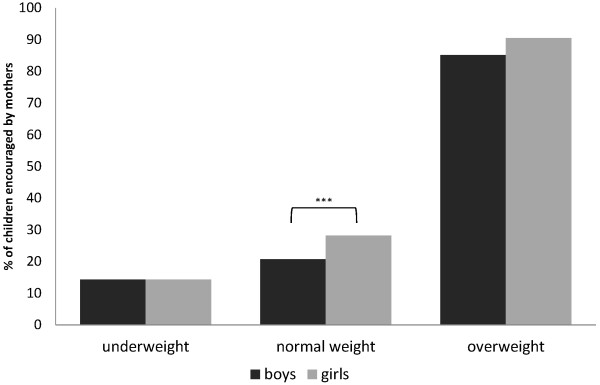
**Maternal encouragement to control weight in under-, normal- and overweight boys and girls.** Comparison of the percentage of mothers of boys vs. mothers of girls encouraging their children to control their weight stratified by children’s weight group. *** *χ*^2^(1)=10.419, p=.001.

Overall, 29% of the sample were encouraged by their mothers to control their weight, girls significantly more often than boys (32.4% vs. 25.6%, *χ*^2^(1)=9.343, p=.002). As shown in Figure 2 this gender difference occurs only in normal weight children. Normal weight girls were encouraged more often than normal weight boys (*χ*^2^(1)=10.419, p=.001), whereas there were no differences between overweight and underweight girls and boys, respectively.

#### Logistic regressions of factors related to maternal encouragement

The variables listed in Table 1 were tested for significant associations (p<.10) with maternal encouragement in univariate logistic regressions separate for boys and girls. Table 2 provides the crude odds ratios and 90% confidence intervals as well as the proportion of encouraged boys and girls in the respective analyses. Logistic regression analyses with stepwise backward elimination were then performed to verify the associations. The resulting model for boys contained child BMI, child migration background, and maternal self-efficacy to promote physical activity as significant predictors of maternal encouragement. In the resulting model for girls child BMI, child migration background, maternal body dissatisfaction and maternal perception of degree of influence on health remained as significant factors. Additionally maternal self-efficacy to promote physical activity remained in the model but lost statistical significance. For the final models shown in Table 3 (boys) and Table 4 (girls) child’s age and maternal education level were added as control variables.

**Table 2:** Possible factors contributing to maternal encouragement of girls and boys to control their weight

	**Boys**		**Girls**	
	**Encouraged**		**Encouraged**	
	**n (%)**	**OR [90% CI]**	**n (%)**	**OR [90% CI]**
**Child characteristics**				
Age, *years*	214 (25.6)	**1.33** [1.09, 1.64]	267 (32.4)	**1.36** [1.12, 1.66]
Migration background, *yes*	203 (25.2)	**2.09** [1.58, 2.77]	261 (32.7)	**2.76** [2.12, 3.58]
Child’s BMI, kg/ *m*^2^	211 (26.0)	**1.87** [1.70, 2.06]	257 (32.1)	**1.84** [1.67, 2.01]
**Maternal characteristics**				
Maternal BMI, kg/ *m*^2^	207 (26.3)	**1.05** [1.02, 1.08]	251 (32.9)	**1.07** [1.04, 1.10]
Tertiary education level	213 (25.8)	**0.54** [0.37, 0.79]	266 (32.7)	1.00 [0.73, 1.37]
Currently smoking, *yes*	207 (25.4)	**1.78** [1.31, 2.42]	260 (32.3)	1.15 [0.85, 1.55]
Active in sports, *yes*	197 (25.1)	0.96 [0.73, 1.26]	253 (32.8)	0.51 [0.39, 0.66]
Body dissatisfaction, *yes*	206 (25.9)	1.20 [0.92, 1.56]	249 (31.9)	**1.76** [1.37, 2.28]
Health consciousness, *high*	211 (25.5)	1.02 [0.78, 1.33]	267 (32.5)	**0.65** [0.51, 0.83]
Perceived degree of influence				
on health, *high*	211 (25.7)	**0.67** [0.45, 0.98]	261 (32.3)	**0.43** [0.30, 0.63]
Self-efficacy to influence child’s				
- TV consumption, *high*	209 (25.3)	**0.67** [0.52, 0.88]	263 (32.2)	**0.60** [0.47, 0.77]
- soft drink consumption, *high*	209 (25.3)	**0.72** [0.55, 0.93]	263 (32.2)	0.80 [0.62, 1.03]
- physical activity, *high*	208 (25.2)	**0.53** [0.41, 0.69]	263 (32.4)	0.52 [0.40, 0.66]

*Note.* n = number of children encouraged to control their weight, OR = odds ratio, CI = confidence interval.

Variables with bolded ORs (indicating p<.10) were included in the subsequent multivariate analyses.

**Table 3:** Final model for boys of factors contributing to maternal encouragement to control their weight

	**B**	**SE B**	**OR**	**[95% CI]**	**p-value**
Child’s BMI, kg/ *m*^2^	.63	.06	1.88	[1.66, 2.13]	.000
Migration background(child), *yes*	.47	.20	1.60	[1.07, 2.37]	.020
Self-efficacy to influencechild’s physical activity,*high*	-.54	.19	0.58	[0.40, 0.85]	.005

*Note.* B = regression coefficient, SE B = standard error of B, OR = odds ratio,

CI = confidence interval.

Final model adjusted for child age and maternal education: N = 767; ***χ***^**2**^(5) = 182.66,

p =.000; -2 Log-Likelihood = 684.87; Nagelkerke’s *R*^2^ =.31; 25.3% encouraged to control their weight.

**Table 4:** Final model for girls of factors contributing to maternal encouragement to control their weight

	**B**	**SE B**	**OR**	**[95% CI]**	**p-value**
Child’s BMI, kg/ *m*^2^	.57	.06	1.77	[1.57, 1.99]	.000
Migration background(child), *yes*	.76	.20	2.14	[1.45, 3.16]	.000
Maternal body dissatisfaction	.46	.19	1.59	[1.10, 2.30]	.014
Perceived degree ofinfluence on health,*high*	-.68	.29	0.51	[0.29, 0.89]	.019
Self-efficacy to influence child’s physical activity,*high*	-.27	.19	0.76	[0.53, 1.10]	.147

*Note.* B = regression coefficient, SE B = standard error of B, OR = odds ratio,

CI = confidence interval.

Final model adjusted for child age and maternal education: N = 724; ***χ***^**2**^(7) = 190.54, p =.000; -2 Log-Likelihood = 711.53; Nagelkerke’s *R*^2^ =.33; 31.5% encouraged to control their weight.

## Discussion

In this study of German primary-school children we investigated related factors of mothers encouraging their children to control their weight. We found that a significant proportion of mothers encouraged their children to control their weight. In accordance with earlier findings in US children and adolescents [12,13], this study found that girls and boys with increasing BMI had a higher chance to be encouraged to control their weight.

One aim of this study was to determine possible gender differences. As expected, girls were more likely to be encouraged to control their weight than boys. However, additional analyses stratified by weight group showed that this was only true for normal weight girls, whereas underweight and overweight girls were encouraged as often as boys. This may be a result of mothers being concerned for both daughters and sons to be overweight but having a greater concern for girls to stay in a normal weight range compared to boys corresponding to the higher pressure on females to be thin. While the encouragement of girls occurred less often if mothers perceived themselves highly able to influence towards health in general, whereas this association could not be seen in regards to boys. Boys, on the other hand, were encouraged less often to control their weight if their mothers report a high self-efficacy to ensure a sufficient level of physical activity in their sons, whereas for daughters this association remained in the final backward regression model but lost statistical significance. This association was also found for girls but did not remain significant in the multivariate regression model suggesting a lower degree of importance of this factor for maternal encouragement of daughters.

According to previous research [7,9], it was also assumed that mothers may be more inclined to encourage their child to control its weight if their own weight is of concern to them. Indeed, results of the current study indicate that mothers who are dissatisfied with their own bodies are more likely to encourage their daughters to control their weight than body-satisfied mothers. For boys, however, there was no such association. This supports previous work which found that the projection of parental weight concerns on to their children’s weight may be more profound in same-gender dyads (e.g. mother-daughter) [16,21].

Additionally, we found a strong association of maternal encouragement with children’s migration background. Children with a migration background had a higher chance of being encouraged to control their weight independent of their mothers’ educational level. Cultural differences may play a role here. A large study in eight European countries showed regional differences in parental concerns about their children’s body weight with parents in Southern Europe reporting more concern about future overweight [22]. Since the majority of migrants in Germany comes from Southern European countries such as Turkey, Italy and Greece [23], this might explain why in this study children with migration background were more likely to be encouraged to control their weight.

The current study has certain strengths and limitations that should be taken into consideration. The associations with maternal encouragement to control weight were studied in a large non-clinical sample of pre-adolescent children from socio-economically diverse backgrounds. This allowed an exploration of the maternal practice to encourage weight-control in daughters as well as in sons. Furthermore, height and weight of the children were measured objectively.Limitations of the current study include self-report of maternal characteristics including height and weight, and the use of single item measures. Also, the cross-sectional design does not allow an interpretation of the direction of the found associations. Due to the self-report nature of the studied variables social desirability or recall bias may have affected the results. Further, some mothers may have interpreted the question about the frequency of encouragement to control weight ("How often do you encourage your child to control its weight?") as an encouragement to gain weight, although considering the usual connotation of weight control, in particular in the context of an overweight prevention programme, this seems unlikely. We used single item measures for the assessment of most of the studied behaviours and cognitions and are thus not able to provide information about reliability or validity of these measures. However, in the context of health assessment it has been shown that single item measures can provide valid information [24].

## Conclusions

In summary, this study found that maternal encouragement to control weight was related to children’s BMI and migration status next to different maternal characteristics depending on the child’s gender. Determining correlates and underlying processes of maternal encouragement can inform preventive measures targeting weight and eating related problems in children. This exploration of possible correlates has demonstrated that not only children’s weight or maternal weight concerns are linked to maternal encouragement to control weight but that mothers’ confidence to influence alternative health behaviours, in this case physical activity, may play a role as well. The results of this study further support the assumption that there are different associations depending on the gender constellation of the parent-child dyad. More research should therefore examine correlates of paternal encouragement to control weight.

## Competing interests

The authors declare that they have no competing interests.

## Authors’ contributions

ACS devised the reported study, collected and analysed data and drafted the manuscript. DK, TW, NE and SK collected data and revised the manuscript. DK also provided advice for the data analysis. JMS is principal investigator of the Baden-Württemberg Study. All authors read and approved the final manuscript.

## Pre-publication history

The pre-publication history for this paper can be accessed here:

http://www.biomedcentral.com/1471-2458/14/450/prepub
